# Community responses to soil remediation for lead exposure reduction in Mirzapur, Bangladesh

**DOI:** 10.1186/s12889-026-28814-y

**Published:** 2026-08-04

**Authors:** Mahbubur Rahman, Syeda Nurunnahar, Jesmin Sultana, Sanjida Adiba, Jenna E. Forsyth, Mitali Das, Gordon K. Binkhorst, Maria Kippler, Rubhana Raqib, Stephen P. Luby, Peter J. Winch, Syed Moshfiqur Rahman

**Affiliations:** 1 Global Health and Migration Unit, Department of Women’s and Children’s Health, Uppsala University, Akademiska sjukhuset, Uppsala, 751 85 Sweden; 2 Environmental Health and WASH, Health Systems and Population Studies Division, icddr,b, Dhaka, Bangladesh; 3 Woods Institute for the Environment, Stanford University, Stanford, CA USA; 4 Pure Earth, Bangladesh Country Office, Dhaka, Bangladesh; 5 Pure Earth, New York, NY USA; 6 Institute of Environmental Medicine, Karolinska Institutet, Stockholm, Sweden; 7 Nutrition Research Division, icddr,b, Dhaka, Bangladesh; 8 Johns Hopkins Bloomberg School of Public Health, Baltimore, MD USA

**Keywords:** Lead, Soil remediation, Qualitative exploration, Community-engaged research, Used lead-acid battery recycling

## Abstract

**Background:**

Lead-acid batteries are widely used for their affordability, availability, and reusability. However, an increase in unregulated and informal recycling, especially of batteries used in electric three-wheelers, has led to widespread environmental contamination in low and middle-income countries. To address this contamination, soil remediation is a proven potential approach that has demonstrated its effectiveness in reducing blood lead levels, but its community responses have not been well documented. This study aimed to explore implementation challenges and achievements, and to assess community perceptions and responses to a soil remediation intervention in Bangladesh.

**Materials and methods:**

A soil remediation intervention was developed and implemented from January through May 2022 in two abandoned lead-acid battery recycling facilities in Mirzapur, a subdistrict in Bangladesh. A qualitative investigation was undertaken in September 2023 to explore the perceptions and responses of residents living near the recycling site and intervention implementers. Respondents were selected using purposive sampling with variation in proximity to the site, exposure to the intervention, and community role. Data were collected through 30 in-depth interviews, 6 key informant interviews, and 2 gender-segregated focus group discussions (*n* = 14). Thematic analysis was performed using an inductive approach.

**Results:**

From the implementation perspective, key achievements included successful soil removal, local labor involvement, and visible environmental improvements, while challenges included limited community outreach, communication gaps, logistical constraints, and a lack of follow-up monitoring. Following remediation, participants reported perceived improvements in crop production, livestock health, and children’s well-being. Risk perceptions were greatly influenced by economic and livelihood concerns, with less emphasis on lead-related health risks. Participants expressed concerns about the safety of soil excavation near homes and the burial of contaminated soil. Key informants acknowledged the need for stronger community engagement, improved communication, and greater transparency to enhance acceptance.

**Conclusion:**

The findings indicate that, although participants perceived environmental and health benefits, gaps in communication, engagement, and transparency in implementation affected overall acceptance. Future interventions should prioritize early community involvement, culturally appropriate communication, and safe waste management systems to improve effectiveness and scalability.

**Supplementary Information:**

The online version contains supplementary material available at 10.1186/s12889-026-28814-y.

## Introduction

Lead is a highly toxic heavy metal with no known safe exposure level. It is particularly harmful to children due to its neurodevelopmental effects [1]. Chronic exposure is linked to a wide range of adverse health outcomes, including reduced IQ, memory loss, developmental delays, respiratory issues, kidney and cardiovascular disorders, and increased risk of premature mortality [2, 3]. These health impacts disproportionately affect populations in low- and middle-income countries (LMICs) where unregulated and unsafe informal recycling practices contribute to widespread environmental contamination. A recent study estimated that over 138,000 cardiovascular disease deaths among adults aged 25 years or older in Bangladesh could be attributed to lead exposure [4]. Lead-contaminated soil is an important pathway of human exposure, especially in communities near informal recycling sites or industrial waste zones [5, 6].

In Bangladesh, the rapid expansion of electric three-wheelers has led to a growing demand for lead-acid batteries. Electric three-wheelers account for 60–65% of the local lead-acid battery demand [7]. Informal used lead-acid battery (ULAB) recycling facilities have proliferated in recent years, releasing hazardous lead dust during manual smelting, breaking, and washing processes [8]. As of 2022, the World Bank identified more than 1,100 informal ULAB recycling sites in Bangladesh, placing an estimated one million people at risk of lead poisoning [9]. Many of these sites operate in densely populated residential and agricultural areas, leading to severe soil and environmental contamination [5]. At one such site in rural Bangladesh, post-assessment studies found that children living within 200 m had median blood lead levels (BLLs) of 19.1 µg/dL—more than five times higher than the CDC’s reference level of 3.5 µg/dL [9].

Globally, soil remediation is considered one of the most effective strategies for reducing environmental lead exposure, usually involving a combination of physical and chemical methods [10, 11]. A traditional remediation approach involves excavating and removing the soil, followed by capping or burial of the scraped soil or landfill disposal, and backfilling the scraped area with clean sand and soil. Recent studies have demonstrated advances in remediation technologies, including the use of ferric sulfate, biochar, and modified mineral-based amendments to reduce lead bioavailability and environmental risk [12–16]. Such interventions have demonstrated success in reducing soil lead levels and improving human health outcomes in limited studies [17].

While technical solutions of soil remediation continue to evolve, much of the literature has prioritized implementation feasibility and engineering effectiveness, with comparatively less attention to the social processes that influence intervention acceptance and sustainability. Recent research emphasizes that community participation is critical for the sustainability of environmental interventions, as it enhances acceptance, promotes behavioral change, and supports long-term maintenance of remediation outcomes [18–20]. Evidence from the U.S. Superfund program, an Environmental Protection Agency initiative designed to identify and remediate hazardous contaminated sites, suggests that insufficient early community engagement may contribute to resistance, misinformation, and reduce intervention effectiveness [21]. For remediation efforts to succeed, community members often need to permit access to contaminated land, participate in awareness activities, adopt recommended protective behaviors, and avoid practices that could lead to recontamination. Transparent communication, culturally sensitive messaging, and repeated community interactions are necessary to build trust and enable behavioral change [22, 23]. Yet, there is limited empirical literature from LMICs on community responses to soil remediation efforts, especially in densely populated rural settings. Understanding how different groups within a community perceive, interpret, and engage with remediation interventions is therefore essential for the effective design, uptake, and sustainability of remediation efforts.

Given the rising threat of lead contamination from widespread informal ULAB recycling in Bangladesh and the need for context-specific evidence, this study addresses a critical gap by exploring community responses to soil remediation interventions. The objectives of the study were to investigate (i) the practical challenges and achievements of implementation, and (ii) community perceptions, concerns, and levels of engagement. Understanding how affected communities interpret and react to such interventions is essential for improving environmental health strategies and informing the design, execution, and scaling of future remediation efforts.

## Methods

### Study design and study site

This descriptive qualitative study employed an implementation science approach to examine how contextual factors, community engagement, and implementation processes influenced both the delivery of the remediation intervention and community responses to it. We conducted in-depth interviews, focus group discussions, and key informant interviews, which were analyzed using inductive thematic analysis and interpreted through an implementation science lens informed by the Consolidated Framework for Implementation Research (CFIR).

**Fig. 1:**
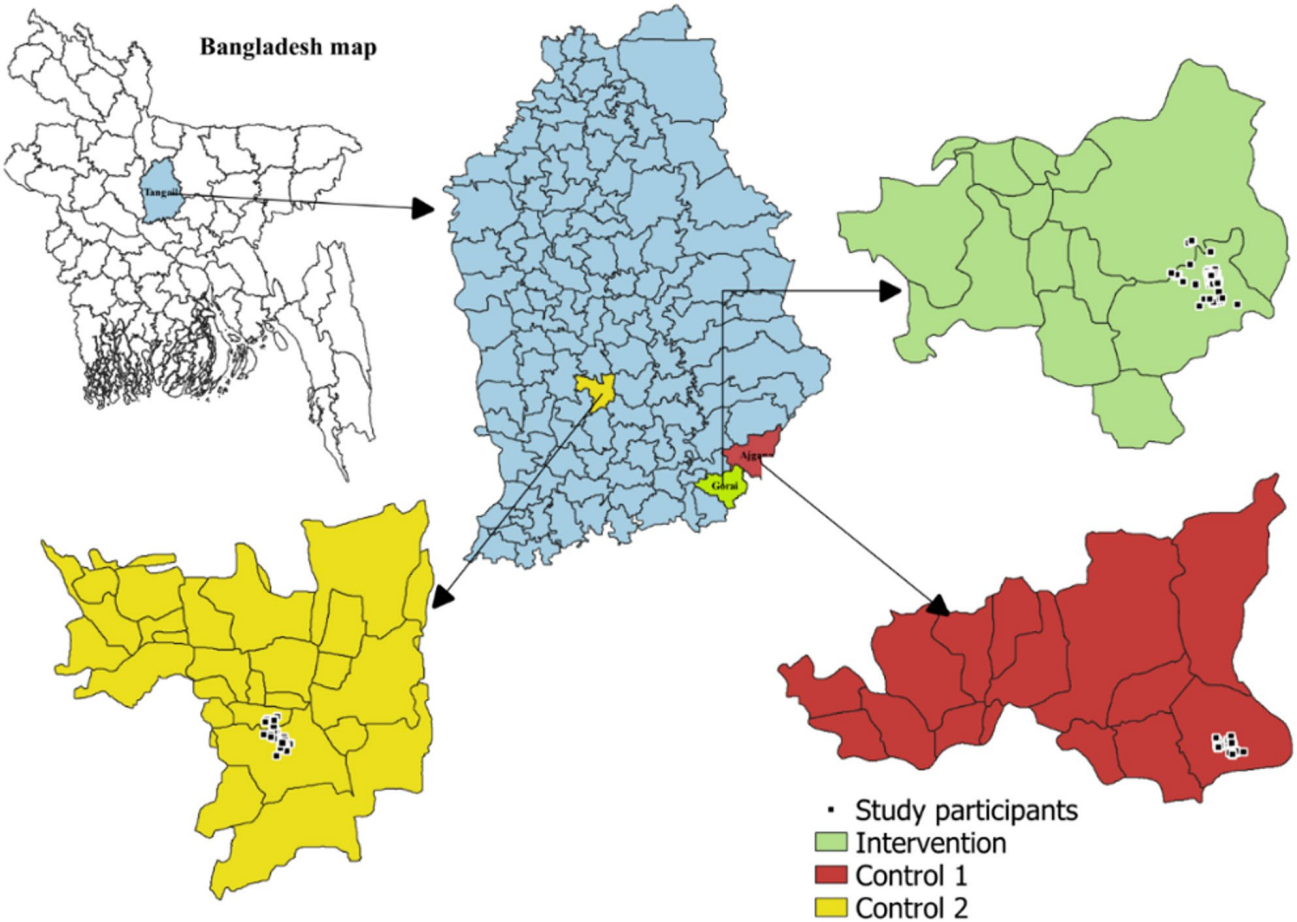
The figure shows the map of Mirzapur with the intervention and control sites. The intervention site was the study site for this study

The study was conducted in a residential area near an abandoned ULAB breaking and smelting facility situated in a rural community in *Mirzapur*, Bangladesh (Fig. 1). The ULAB recycling site operated in this residential area for three and a half years before closing in February 2019. This site was identified as a priority site for remediation in the Toxic Site Identification Program (TSIP) database by Pure Earth Bangladesh. Both residential and agricultural lands adjacent to or near this site were contaminated with lead waste and lead-laden plastic waste. The area identified for soil remediation and cleaning was approximately 45,000 square meters (4.5 hectares). Intervention activities started in January 2022 and ended in May 2022, while qualitative data collection was conducted in September 2023. This delay of almost 16 months increases the chances of poor recollection and recall bias regarding specific operational details of the intervention. However, it also allowed the participants time to reflect on perceived long-term changes, lingering concerns, and their overall experience of the intervention.

### Intervention overview: soil remediation process

**Fig. 2:**
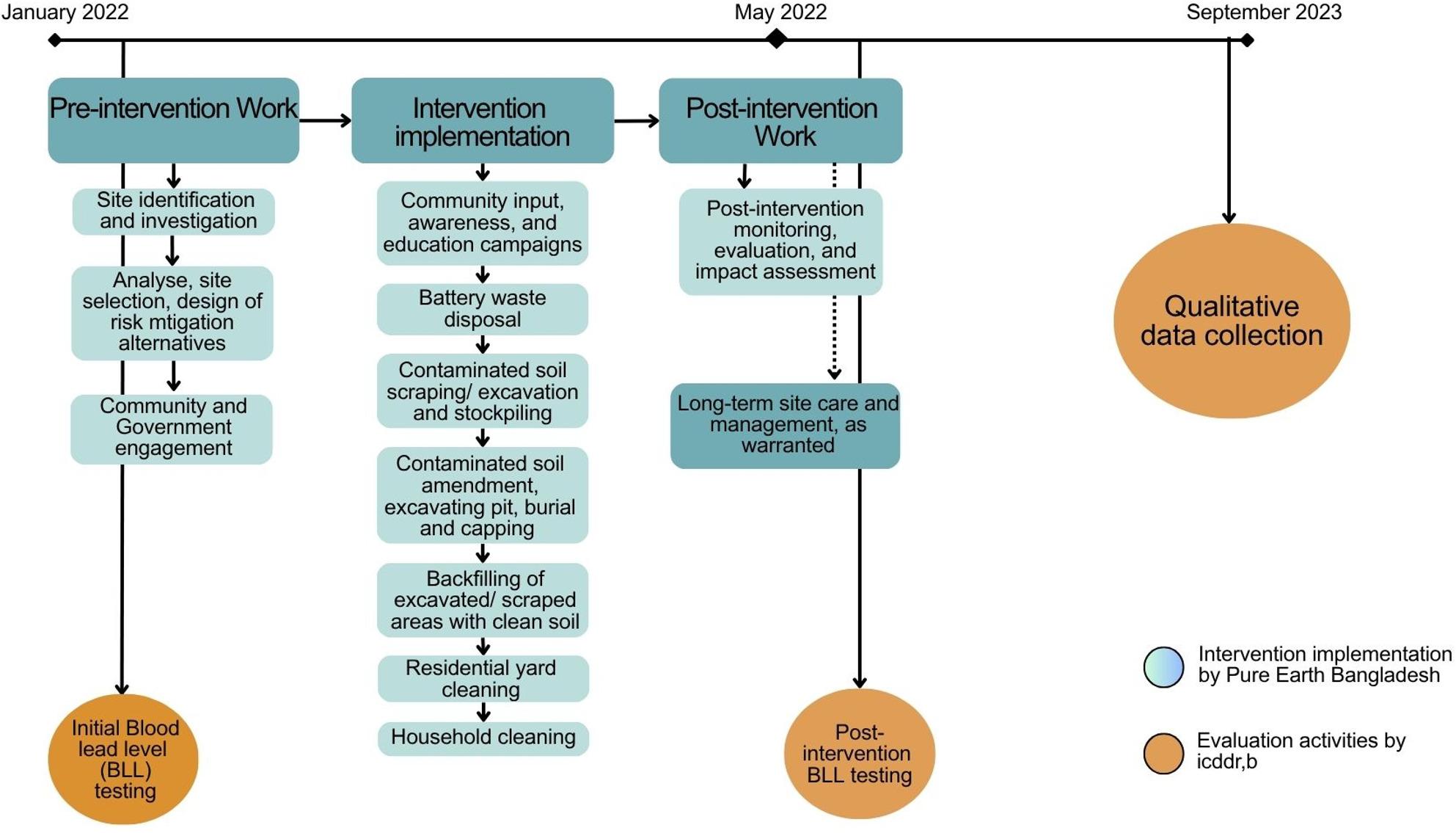
Flow chart showing the soil remediation process and evaluation activities

The intervention included a sequential set of activities, including initial and detailed site assessment, community consultation, soil excavation, refilling, contaminated soil disposal, household cleaning, and health awareness efforts. Figure 2 presents a visual overview of the intervention process from planning through implementation. About 600 cubic meters of scraped soil and leaf matter were treated and buried, approximately 8,200 kg Triple Superphosphate (TSP) was applied during soil treatment, and around 14.4 cubic meters of residual ULAB waste were collected and disposed of at a municipal landfill. Two burial pits were also constructed and capped with at least 1 m of clean soil. The remediation intervention was implemented by Pure Earth Bangladesh, in collaboration with the Department of Environment for monitoring and supervision, and with field-level implementation by the Geology Department of Dhaka University. The research and evaluation were conducted by International Centre for Diarrhoeal Disease Research, Bangladesh (icddr, b), through assessments of blood lead levels and qualitative data collection on community responses.

### Study population

The study population consisted of residents living in the vicinity of the ULAB recycling site in *Mirzapur*, who were either directly exposed to the intervention (i.e., their homes were cleaned or soil was excavated near their property) or indirectly affected (i.e., attended meetings or lived within 200–400 m of the site).

### Study participants and data collection method

**Fig. 3:**
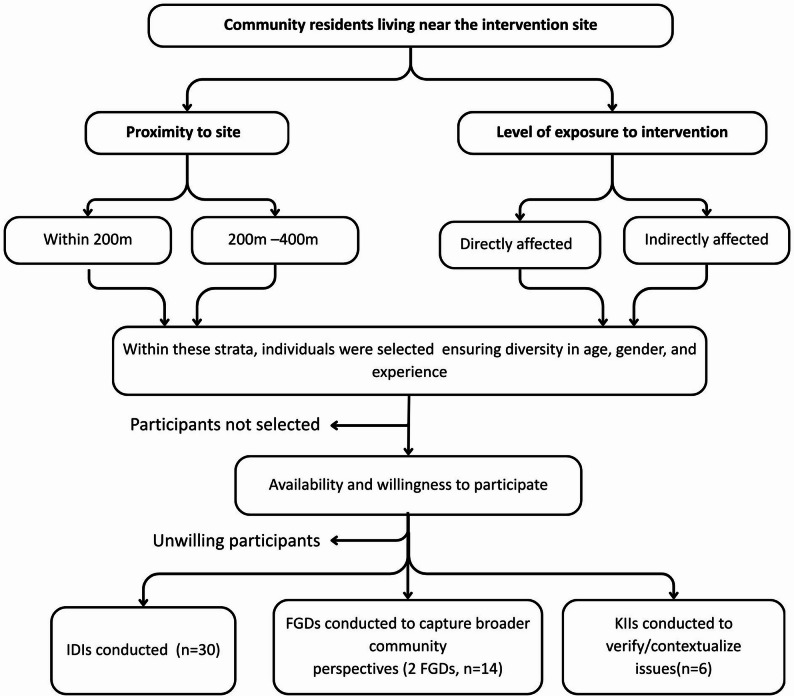
Diagram showing the steps of stratified sampling

Data were collected through in-depth interviews (IDIs), focus group discussions (FGDs), and key informant interviews (KIIs). These methods were used together to capture individual experiences, collective community perspectives, and different aspects of the implementation process. The participant selection process for IDIs, FGDs, and KIIs is summarized in Fig. 3. A total of 30 IDIs were conducted using semi-structured one-to-one interviews designed to explore participants’ personal lived experiences and perceptions in depth. Participants for FGDs and in-depth interviews were community residents recruited using purposive sampling, with selection based on proximity to the contaminated site (e.g., within 200 m and 200–400 m), the extent of direct interaction with remediation activities, and roles within the community, while ensuring diversity in age, gender, and experience.

Two FGDs were conducted with 14 participants (seven men and seven women), organized into gender-homogeneous groups. Gender was used as a grouping variable to create a safe environment for open dialogue in the rural Bangladeshi context, where gender norms influence the comfort, participation, and willingness to speak freely in mixed groups. This also allowed exploration of gender-specific experiences and perceptions related to the intervention and engagement with the remediation process. Participants were encouraged to reflect on shared experiences, attitudes, and local narratives about the soil remediation process.

In total, six KIIs were conducted to complement community perspectives by providing insights into implementation processes and contextual factors. Key informants were selected through purposive sampling based on their leadership roles in the community, or technical expertise and involvement in the intervention. This included community leaders, religious leaders, local political representatives, and implementers. These interviews provided insights into decision-making processes, implementation challenges, and contextual dynamics that community participants had not fully captured.

In-depth interviews lasted for 45–60 min, key informant interviews lasted for 30–45 min, and FGDs took more than an hour. The interview guides were developed by the research team to explore themes such as perceptions of contamination and cleanup, communication quality, trust in the implementers, observed outcomes, and recommendations for future projects. The interview guides are provided as supplementary files 1, 2, and 3.

A trained qualitative research team and investigator from icddr, b conducted all interviews and group discussions. This separation of the implementation and evaluation teams helped reduce potential influence and bias in the intervention’s success during data collection. All data collection sessions were conducted in Bengali (the local language) and took place in neutral, quiet settings such as household courtyards or indoor private spaces to ensure participant comfort and confidentiality. Sessions were audio-recorded with participants’ consent and transcribed verbatim. Transcripts were then translated into English, with local terms and cultural references preserved. To ensure accuracy and preserve contextual meaning, translated transcripts were cross-checked against the original Bengali transcripts by members of the research team. Any discrepancies were discussed and resolved through consensus, with particular attention to culturally specific terms and expressions.

Recruitment and data collection continued until thematic saturation was reached, defined as the point at which no new themes, concepts, or perspectives emerged from the ongoing analysis of interviews and focus group discussions [24, 25]. The research team held regular debriefing sessions and reviewed transcripts to confirm saturation before concluding data collection. This iterative process ensured the adequacy and depth of the sample to address the study objectives.

### Analysis

Transcripts from IDIs, FGDs, and KIIs were treated as a single body of data and were initially analyzed thematically using an inductive approach, following the principles outlined by Bernard [26]. Thematic analysis identified patterns and concepts emerging directly from the data, without applying a predefined framework. Two members of the research team with training and experience in qualitative research independently reviewed the transcripts and manually generated initial codes based on close reading and annotation.

During analysis of the focus group material, attention was paid to group dynamics, including areas of consensus, shared experiences, and points of disagreement among participants. Instances where participants reinforced, expanded upon, or challenged one another’s statements were noted, as these interactions helped contextualize and refine emerging themes. After coding independently, the researchers grouped related codes into broader thematic categories aligned with the study’s objectives. Any discrepancies were resolved through regular discussions to ensure consistency and reflexivity, and shared interpretation [27, 28]. To enhance analytic rigor, the themes were refined through peer review, team discussions, and triangulation with field notes.

**Fig. 4:**
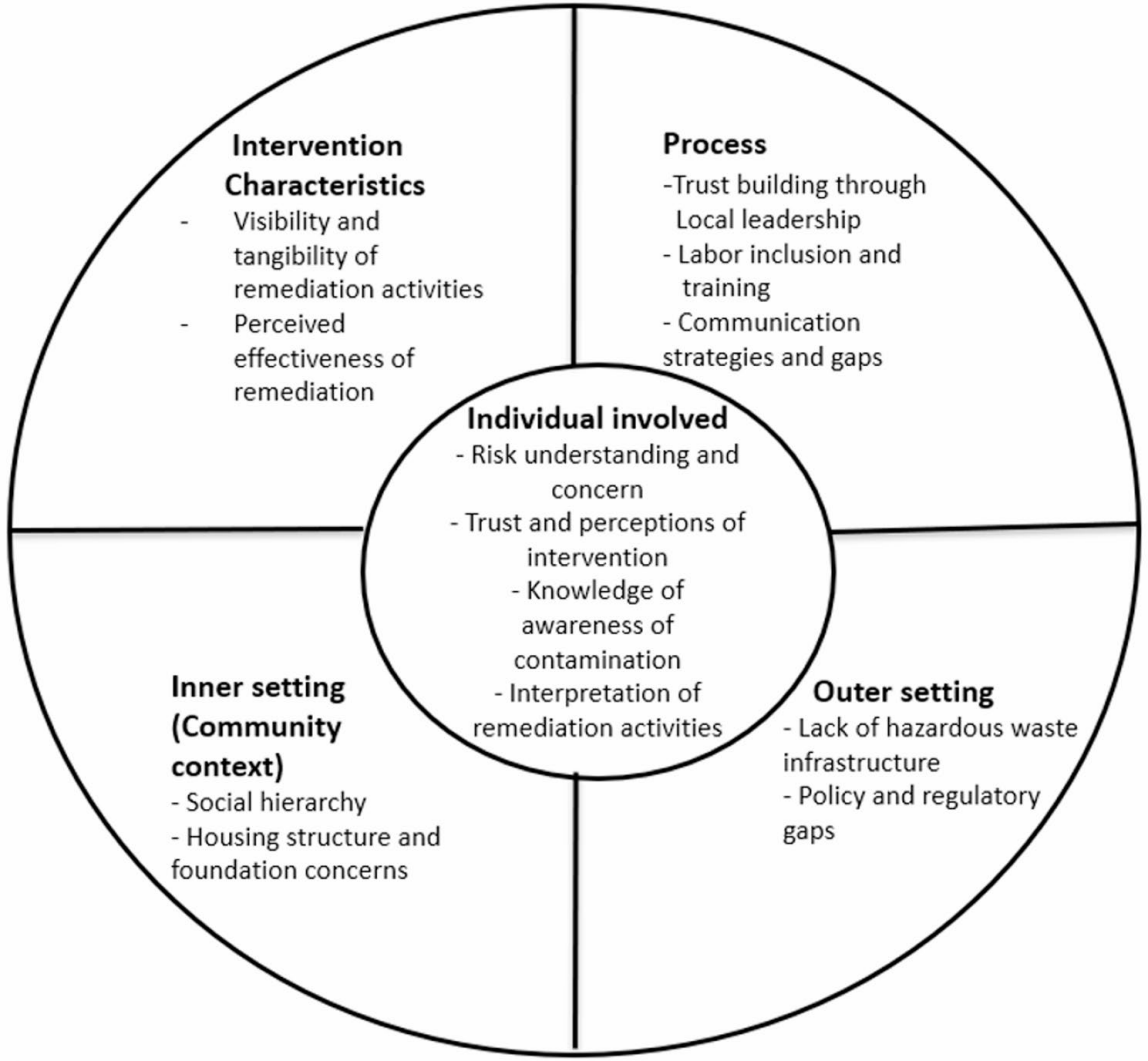
Conceptual layout adapted from the consolidated framework for implementation research (CFIR) from Damschroder et al (2009)

To support interpretation of findings, results were examined using the Consolidated Framework for Implementation Research (CFIR) [29]. CFIR is a framework that helps guide the exploration of the reasons why implementation efforts succeed or face challenges by directing attention towards five areas: (1) the intervention itself (2), the outer setting (3), the inner setting, 4) the people involved, and (5) the implementation process. CFIR was applied to the inductively coded data to further organize and interpret the findings. Figure 4 presents the conceptual mapping of study findings against the CFIR framework.

## Results

The findings are organized in line with the study objectives, (i) reflecting implementation-related challenges and achievements, and (ii) residents’ perceptions, concerns, and levels of engagement.

### Characteristics of study participants

Table 1 illustrates the characteristics of study participants (*n* = 50) across in-depth interviews, focus group discussions, and key informant interviews. Most participants were over 30 years old. Women were more frequently represented in the in-depth interviews, while all key informant interview participants were men. Participants had varied educational backgrounds, with many reporting secondary-level schooling. The majority of participants came from small households (fewer than five members), a pattern especially noted among key informants.

**Table 1:** Characteristics of participants

Variable	Category	In-depth Interviews (*n* = 30) *n*	Focus Group Discussions (*n* = 14)	Key Informant Interviews (*n* = 6) *n*
*Age of respondent*	< 30 years	10	5	-
	> 30 years	20	9	6
*Gender*	Female	19	7	-
	Male	11	7	6
*Education*				
	No education (0)	2	1	-
	Primary	6	6	1
	Secondary	14	7	1
	Higher secondary	7	-	4
	Above higher secondary	1		
*Number of family members in household*	< 5	23 7	-	6
	> 5			

### Implementation in practice: challenges and success

This theme addresses the first study objective by exploring practical challenges and achievements of implementation. As summarized in the coding tree Table 2, the findings are organized into four interrelated subthemes that reflect key aspects of implementation: trust-building and initial reactions, community involvement and labor inclusion, communication processes, and logistical and technical constraints. Together, these subthemes describe how social, operational, and contextual factors shaped the implementation process.

**Table 2:** Theme, subthemes, categories and codes derived from inductive thematic analysis

Theme	Subtheme	Category	Codes
THEME 1 Implementation in practice: Challenges and successes	Trust and acceptance	Initial perceptions and fear	Rumors of land acquisition
			Fear of private interests
			Prior negative industrial experiences
		Trust-building mechanisms	Chairman’s reassurance
			Involvement of respected leaders
			Repeated interpersonal engagement
	Community involvement and participation	Local labor inclusion	Employment opportunities
			Pride in participation
			Economic benefit
		Recognition of intervention effort	PPE use as sign of seriousness
			Visible machinery
			Professional conduct of workers
		Communication processes	Information dissemination
			Leaflet distribution gaps
			Mosque announcements
			Lack of prior notification
		Barriers to engagement	Seminar timing
			Religious norms
			limited reach to tenants
	Logistical and technical constraints	Waste disposal challenges	Absence of hazardous waste facilities
			Long-distance transport
		Land negotiation challenges	Landowner reluctance
			Stigma and fear of devaluation
THEME 2 Perceived benefits and concerns after remediation	Perceived benefits	Environmental improvements	Crop recovery
			Odor reduction
		Livelihood and health outcomes	Livestock recovery
			Perceived child health improvement
	Community concerns	Excavation quality	Insufficient depth
			Uneven excavation
		Structural safety	Soil cohesion loss
			House foundation cracks
		Sustainability and follow-up	Lack of monitoring
			Absence of BLL testing

#### Building trust and navigating suspicion

Several community participants mentioned an initial sense of confusion, fear, or suspicion regarding the intervention’s intentions. Despite early efforts by implementers to introduce the project and engage with residents, misunderstandings were reported, especially during the initial stages. Fears were linked to rumors about land acquisition or private interests. As one of the key informants said,

*At first, people didn’t understand what was happening. They were afraid a company might come and take their land or cause harm.*

*(KII_02)*

These concerns appeared to stem from prior negative experiences with industrial activity in the area. Community residents reported that clarification was only achieved after trusted local leaders intervened and provided assurance about the nature of the project. The involvement of the *chairman (elected community representative)* and other respected individuals was repeatedly cited as a pivotal factor in fostering community acceptance. As one of the key informants opined,

*Without the chairman and local leaders, no one would have agreed. It took a lot of convincing.*

*(KII_03)*

An implementer acknowledged that building trust required sustained interpersonal engagement beyond technical planning and documentation. 

#### Community involvement, labor inclusion, and recognition of efforts

Community involvement through the recruitment of local residents as workers for remediation activities emerged as a notable strength of the intervention. Workers were recruited locally through a labor-supplying agency. Many participants expressed appreciation that young men from their neighborhood were directly involved in remediation tasks, such as excavation, transportation of contaminated materials, and refilling yards with fresh soil. This local participation was perceived as beneficial both economically and socially. As one of the participants mentioned,

*Young men from our neighborhood worked on the project. They wore work dress (PPE), and we were happy they got this opportunity. *

*(IDI_06)*

The use of personal protective equipment (PPE) by workers and the visible presence of technical tools, health and safety signage, and machinery were interpreted by some residents as indicators of the seriousness and potential hazards of the work being conducted. One of the participants expressed,

*They came with gloves and masks, so we thought it must be something dangerous. *

*(IDI_R5)*

Several participants also recognized and appreciated specific intervention activities, including the removal of visibly contaminated material, yard cleaning, and refilling of soil, which they associated with improved environmental conditions. One of the participants whose household was cleaned during the intervention said,

*They cleared our yards and filled them with clean soil. It looked much better than before.*

*(IDI_R12)*

Implementers noted that, while local labor inclusion facilitated field operations and community rapport, it also required additional efforts to provide training and ensure proper health and safety practices. According to the implementer, hired workers underwent three training stages: (i) verbal training, (ii) safety training including the importance of using personal protective equipment (PPE) such as gloves, masks, safety boots, and (iii) training on machinery operation. Workers were divided into sub-groups and closely monitored by the implementers. 

#### Communication gaps and missed opportunities

Several participants reported having limited or no prior knowledge of the project before activities began. While implementers used leaflets and mosque announcements to disseminate information, many community members reported not receiving or reading the leaflets, and some were unaware of key engagement events, such as the community seminar. Since most activities were concentrated on the contamination site, the outreach was limited. Some key informants mentioned the low attendance at the seminar and recommended scheduling future seminars on weekends to increase participation. One key informant explained,

*There have been seminars, but I haven’t heard or seen anything myself. However, if this effort remains confined, it won’t suffice. Awareness is the key. If the seminar had been held on a holiday or a weekend, many people would have attended. *

*(KII_04)*

One of the key informants did not attend the seminar, despite being contacted as a stakeholder, because of his religious beliefs. As he said,

*A seminar was held here. I don’t know what was discussed in the seminar. They told me to attend, but I didn’t go. The reason I didn’t go was that they had brought in musical performers, and I felt that if it went against my religious beliefs, I shouldn’t attend. As an Islamic scholar and Imam, it wouldn’t be appropriate for me to go. Everyone should respect that, and even if others don’t, I have to adhere to it. *

*(KII_03)*

Several participants emphasized the importance of directly notifying both tenants and landowners before the intervention began to ensure greater community awareness and participation. 

#### Logistical challenges and technical constraints

From the implementers’ perspective, the intervention was accompanied by some logistical and infrastructural challenges. Site selection emerged as one of the most complex tasks, requiring a careful balance between contamination severity, population density, and accessibility. In addition, the transportation and disposal of lead-contaminated scrap and soil presented further difficulties. Due to the absence of a designated hazardous waste disposal facility in the area, the team was compelled to adopt a temporary solution. Ultimately, the waste had to be transported to a municipal site located a considerable distance from the intervention area. A key informant from the implementation team described this challenge, stating,

*We collected around 8 tons of battery waste from the Mirzapur site,* *but the disposal was challenging.* *There’s no* *formal hazardous* *waste station.* *However, the scraps* *were dumped* *at the Kaliakair* *waste management facility,* *14* *km away*.

*(KII_K6)*

To facilitate safe waste burial, a landfill pit was created approximately 500 m from the contaminated site. However, securing land for this purpose was not straightforward. Negotiations with local landowners proved difficult, as many were hesitant to offer their land due to fears of contamination stigma and potential devaluation. As one key informant from the implementation team explained,

*Many didn’t want to give their land, fearing its value would decline*.

*(MKII_K6)*

Despite these challenges, the land was eventually secured for remediation through extended negotiation and engagement with local leaders and authorities.

### Residents’ perceptions, concerns, and levels of engagement

This theme addresses the second study objective by capturing residents’ perceptions, concerns, and levels of engagement with the intervention. As shown in Fig. 2, participants reported a range of perceived benefits, including improved health of children, livestock, and crops, as well as lingering concerns about the quality of implementation. The subthemes presented below reflect how residents evaluated the intervention’s impacts while also expressing uncertainties. 

#### Perceived benefits of the intervention

The majority of the participants receiving the intervention perceived an improvement in livestock health following the soil remediation intervention. Although many participants observed improvements in livestock health, some still noted incidents of animal deaths, suggesting the presence of residual contamination or other environmental factors. According to the implementer, after the recycling factory began operating, villagers observed an increase in livestock deaths in the area, which they attributed to contamination from the recycling activities. As a result of the intervention, lead-laden grasses and bushes near the village were uprooted from the contaminated soil of fields and yards, which protected livestock, birds, and other Several participants perceived improvements in plant health as well, attributing these changes to restored soil fertility due to the soil remediation intervention. Participants shared that, before the intervention, leaves showed discoloration, brown spots, and shredding, which they associated with contamination from the nearby lead battery recycling factory. They perceived that the growth of vegetables and fruits seemed to have stagnated since the establishment of the recycling factory in this area. One of the participants opined,

*Previously, people in this area suffered a lot because vegetables couldn’t grow due to the contamination from the sishar karkhana (battery factory), but now, after cleaning up that area, vegetables are growing again *

*(IDI_R4)*

Residents mentioned that they observed satisfactory results from farming, which they associated with the cleaning of yards and refilling with clean and fertile soil. Women were especially happy, as fruits began growing again in a healthy manner after the intervention.

Implementers believed that children’s health was significantly affected by lead pollution as they frequently played near the piles of lead waste discarded from the ULAB facility, unaware of the health hazards. A resident who lived near the factory during her pregnancy shared,

*My elder son was in class 1 when the ULAB started operating in our neighborhood. After that, my son suddenly started showing signs of memory loss and learning difficulties. *

*(IDI_R3)*

The majority of participants perceived improvements in child health, which they primarily described in terms of general child health, not attributing them to reduced lead exposure. Some participants highlighted the role of behavior changes, such as wearing shoes outside, washing hands and feet after coming indoors, and adopting a healthier diet, in further enhancing child health. Traditionally, many rural residents walked barefoot inside the house, yards, and fields, increasing direct exposure to contaminated soil.

The majority of participants reported experiencing persistent foul odors in the area before the intervention. Some tenants even left their homes because of the bad odors. Some participants specifically complained about an intolerable acidic odor, particularly during the rainy monsoon season. Although lead is odorless, strong acids used in ULAB recycling create fumes with a distinct astringent smell. Participants in focus group discussions expressed relief that the unpleasant odor had dissipated after the intervention. A female participant said,

*There was always a bad smell outside the house. However, the smell disappeared after the soil replacement. *

*(IDI_R2)*

#### Community concerns

Community members expressed several concerns about how the intervention was carried out. These concerns primarily centered on the adequacy of soil excavation and the quality of the refill material.

Male participants in the focus group discussion felt that the soil had not been excavated deeply enough, and some respondents in the in-depth interviews echoed this perception. As one participant stated,

*The topsoil was supposed to be excavated up to one (1) foot, but only six (6) inches were cut. If it had been dug one foot deep, the acid level would have been lower, resulting in a reduced harmful impact of lead. The soil is still not completely free of lead, and this remains a cause for concern. *

*(FGD_1)*

Additionally, participants expressed concerns about the type of refill material, noting that sand appeared to be used instead of solid soil. Some worried that excavation could weaken soil cohesion around houses, potentially affecting foundations, particularly during heavy rains. A family living very close to the recycling facility shared their concern, saying:

*My house’s foundation has developed cracks because the soil’s cohesion weakened due to being overturned by the excavator machine*

*(IDI_R6)*

This structural vulnerability was a major concern for residents, particularly because many one- and two-story buildings in the area lack deep foundations. Some participants also perceived that the amount of refilled soil was less than expected. A key informant similarly noted that residents had voiced such concerns, supported by saying,

*They (implementers) didn’t dig the amount of soil they told us and didn’t give the appropriate amount of clean soil. They skipped some of the places in low-density areas. *

*(KII_01)*

Some key informants spoke about the absence of post-remediation follow-up activities, such as monitoring of the buried pit, return visits by the implementation team, or periodic blood lead level assessments. They expressed that periodic follow-up would strengthen the intervention and help prevent recontamination. Though an implementer noted that a post-investigation was conducted in June 2023 to assess soil and environmental conditions one year after remediation, this was not reflected in the interviews.

## Discussion

The implementation of a soil remediation intervention in Mirzapur was influenced by both technical execution and community-level processes. Although the intervention was perceived as beneficial, its implementation revealed challenges related to engagement, communication, infrastructure, and governance. Community responses underscored the role of social relationships, visible remediation activities, and local participation in shaping acceptance, alongside ongoing concerns about implementation quality and sustainability.

To interpret these findings, we draw on a conceptual framework informed by the Consolidated Framework for Implementation Research (CFIR), which emphasizes how implementation outcomes are shaped by interactions between implementation processes, community context (inner setting), and broader structural conditions (outer setting). This framework provides a lens for understanding how trust, communication practices, and infrastructural constraints influenced community responses to the intervention.

The Mirzapur intervention followed a structured, stepwise implementation process that aligns with established remediation frameworks such as the U.S. Superfund process, including site assessment, planning, implementation, and, critically, community involvement [30]. From a CFIR-informed perspective, these activities reflect key implementation “process” components, particularly engaging local actors and executing visible remediation tasks. In Mirzapur, local labor recruitment not only facilitated field operations but also fostered a sense of ownership and temporary economic benefit for residents, echoing observations from other rural remediation contexts where local employment increases community investment in project success [31].

Despite these similarities, the intervention differed from the Superfund model in important ways. In Superfund projects, the Environmental Protection Agency (EPA) prioritizes well-informed community participation from the outset, with opportunities for input on cleanup decisions [30]. In Mirzapur, awareness activities primarily reached landowners and households within 200–400 m of the former ULAB site, while some residents reported learning about the project only after activities began. In settings where printed leaflets may not reliably reach or engage all households, more direct communication methods such as courtyard meetings, mosque loudspeaker announcements or tenant landowner notification could have improved the reach and participation.

The adopted communication approach limited the opportunities for early engagement, which hampered trust-building for some residents, a finding consistent with research highlighting the pivotal role of transparent, inclusive communication in environmental health interventions [22, 32, 33]. The results also reveal how logistical and cultural factors shaped community participation. In addition, individual cultural or religious preferences occasionally influenced attendance, as illustrated by one participant who declined to attend due to concerns about musical elements of the event. Similar to findings from other environmental and health interventions, cultural factors, such as religious norms and timing of activities, influenced inclusivity, suggesting that more tailored outreach could further strengthen participation [33]. These challenges underscore the significance of the community’s internal heterogeneity and dynamics, encompassing social hierarchies, religious norms, and patterns of information exchange, in shaping participation and trust. Global best practices recommend engaging religious leaders, aligning activities with local rhythms such as prayer times or holidays, and co-developing materials with community stakeholders to enhance buy-in and ensure that no groups are unintentionally excluded [34].

Communication gaps also influenced perceptions of the intervention’s effectiveness. Within the CFIR-informed framework, these gaps reflect limitations in implementation processes related to communication and engagement. Concerns about excavation depth, refill materials, and the scope of the work shaped community perceptions and, in some cases, created uncertainty about the intervention’s effectiveness, regardless of whether these concerns reflected actual technical limitations or misunderstandings of remediation procedures. Participatory planning, early household-level consultations, and clear explanations of technical processes and strategies recommended by the World Health Organization and other agencies [34] can help strengthen community understanding and confidence in remediation activities.

Residents’ risk perceptions were also distinct. Although they recognized improvement in child and livestock health, they often interpreted environmental risk through economic and livelihood lenses, which were more immediate and tangible to them. Quantitative evaluation of the intervention demonstrated a reduction in children’s blood lead levels [35]; however, participants in this study did not explicitly attribute these improvements to reduced lead exposure, suggesting a gap between measurable exposure reduction and community understanding of lead-related health risks. This pattern is consistent with broader evidence showing that communities often interpret environmental health risks through observable impacts on daily life and livelihoods rather than abstract biomedical indicators [36]. This pattern underscores how local knowledge systems and livelihood priorities within the community’s inner setting shape risk interpretation, reinforcing the need for context-sensitive risk communication strategies. Risk communication strategies should connect scientific measures of exposure to locally salient outcomes. Limited awareness of lead’s long-term health effects, particularly for children, underscores the need for targeted risk communication that links environmental hazards to locally relevant concerns and provides practical guidance on protective behaviors.

Key informants noted the lack of post-remediation follow-up activities, such as pit monitoring, household visits, or periodic child BLL assessments, and highlighted this as an area that could strengthen future interventions. From an implementation science perspective, the absence of structured post-remediation follow-up represents a gap in sustaining implementation gains, which is critical for long-term effectiveness. In the Superfund model, post-construction completion activities like operation and maintenance, as well as formal site closure procedures, help sustain remediation benefits and ensure long-term protection [30]. Introducing similar follow-up measures in contexts like Mirzapur could help maintain remediation outcomes and reduce the risk of recontamination over time.

The intervention also faced significant infrastructural challenges. Bangladesh currently lacks dedicated hazardous waste disposal facilities, forcing implementers to adopt temporary solutions such as transporting contaminated material to a depot 14 km away. While functional in the short term, such ad-hoc arrangements do not address systemic waste management. These constraints reflect outer setting factors within the CFIR-informed framework, particularly policy and infrastructural limitations that shape what implementers were able to achieve locally. Findings highlighted the urgent need for legislative and infrastructural reforms to support safe, sustainable hazardous waste disposal.

These findings underscore that remediation is not only a technical exercise but also a social and political/governance issue shaped by trust, cultural alignment, communication, and infrastructure. Addressing these social dimensions alongside technical challenges is key to ensuring that soil remediation efforts are both effective and sustainable in lead-contaminated areas.

The study has a few limitations, including the potential social desirability bias and retrospective recall bias, as interviews were conducted approximately 1.5 years after the intervention. However, this time interval may also have allowed participants to reflect on longer-term experiences and perceived impacts of the intervention. Future studies could benefit from incorporating multiple qualitative time points to capture evolving perceptions over time. The absence of pre-intervention qualitative data also limits the ability to compare community perceptions before and after remediation. However, qualitative research primarily explores perceived changes and explanatory narratives rather than establishing causal attribution. In this study, participants’ accounts provided valuable insight into how community members understood and interpreted changes associated with the intervention. Despite these limitations, the credibility of the findings was strengthened through triangulation of the data across respondent groups. By methodically documenting every phase of data collection and analysis, we sought to ensure dependability. And, to minimize potential research bias, interpretations were grounded in participants’ verbatim quotes and regular peer debriefing.

## Conclusions

This study demonstrates that the effectiveness of a soil remediation intervention in lead-contaminated settings is shaped not only by technical execution but also by the quality of community engagement throughout the implementation process. Findings from both implementation experiences and community responses indicate that gaps in early communication, limited outreach, and a lack of transparency influenced trust and shaped perceptions of the intervention quality. Scaling such interventions across high-risk contexts in Bangladesh will require coordinated efforts from government and non-government actors, rooted in participatory, equitable frameworks that empower local voices and address structural barriers to environmental health.

## Future perspective

Building on these findings, future interventions should prioritize the following key areas: (1) community-centered engagement strategies (2), culturally attuned awareness-building (3), infrastructure for safe hazardous waste disposal, and (4) continued health monitoring, including periodic BLL assessments. In addition, complementary remediation approaches that stabilize or reduce lead contamination while minimizing large-scale soil excavation may help address community concerns related to household disruption, livestock, and agricultural livelihoods during scale-up. Finally, sustainable prevention of lead contamination will require stronger regulation of informal battery recycling practices and a gradual transition to safer battery technologies. 

## Supplementary Information

Supplementary Material 1.

Supplementary Material 2.

Supplementary Material 3.

## Data Availability

The deidentified data can be made available upon reasonable request to the corresponding author.

## Abbreviations

CDC: Center for Disease Control and Prevention
CFIR: Consolidated Framework for Implementation Research
LMICs: Low- and middle-income countries
ERC: Ethical Review Committee
RRC: Research Review Committee
TSIP: Toxic Site Identification Program
TSP: Triple Superphosphate
ULAB: Used Lead Acid Battery
