# Supplementary material for: Community responses to soil remediation for lead exposure reduction in Mirzapur, Bangladesh
Source: BMC Public Health. 2026 Aug 4;26:2370. doi: 10.1186/s12889-026-28814-y (PMC13476908; doi:10.1186/s12889-026-28814-y)
Supplement: Supplementary file 2 [file 12889_2026_28814_MOESM2_ESM.docx]

# Supplementary File 2: Focus group discussions with the community members

# Study title: Community Responses to Soil Remediation for Lead Exposure Reduction in Mirzapur, Bangladesh

# Focus group discussions with the community members

**Study Title: Effectiveness of soil remediation of abandoned ULAB sites, nearby household cleaning, and community awareness intervention to reduce lead exposure among children**

**IRB/ERC No.: PR-21123**

**Version Number/Date:**

*Unique ID #:*

*Location and Date:*

*Facilitator:*

*Start Time:*

*End Time:*

Goal:

- Understand perceptions of the intervention among the community members
- Assess the acceptability of the intervention among the community members

(*Note for the interviewer: We want to know what they call the “intervention” in Bangla. How do they refer to it. Therefore, we should not put words in their mouths. We want to start by learning their words. We should start the questions in a more open-ended way, so that we can elicit their words. Do not introduce the words “soil remediation” or “intervention” or “lead” or “lead education” unless the study participants themselves use these words.)*

Q1. What kinds of health problems/diseases can people or animals get from contact with the soil? What types of problems might there be in the soil?

*(Note for the interviewer: Try to get their words for the problems. Do not introduce words like ময়লা and সীসা unless they first say the words. Do not say “Why is there ময়লা in the soil?” or “Tell us about the intervention to remove সীসা from the soil…”. We want to first hear what they say, how they describe it.)*

Q2. Did some people come here to your community to clean up the area? Who were they? Where did they come from?

Q3. Please tell me (give me a list of) the different things they did.

Q4. Please describe each of the things they did:

| Activity | Describe the activity | Who did it? What equipment did they use? | How long did it take? | Any special words to label it, to call it |
| --- | --- | --- | --- | --- |
| 1 |  |  |  |  |
| 2 |  |  |  |  |
| 3 |  |  |  |  |
| 4 |  |  |  |  |

Q5. [Write the names of the different activities on cards. Or draw a small picture of each activity on each card] Okay, here are some cards that show the activities you just told me about. I would like you to put them in order, from the activity that you think was most important / beneficial for the community, to the activity you think was least important.

- [Show the card for the most important activity]. Why was this the most important activity? What were its benefits for the community? What were the problems with this activity?
- [Show the next card]. Why was this the next most important activity? What were its benefits for the community? What were the problems with this activity?
- [Show the next card]. Why was this the next most important activity? What were its benefits for the community? What were the problems with this activity?
- [Show the next card]. Why was this the next most important activity? What were its benefits for the community? What were the problems with this activity?

Q6. Thinking about all of these activities, what word do you use to refer to them? Do people use any other words for it?

Q7. What recommendations do you have for [use local term] activities?

Q8. What did you find most memorable about the [use local term] you received?

Q9. What have you and your family members changed because of the intervention?

Q10. What was difficult to change?

Q11. What recommendations do you have to make it easier to adhere to the [use local term] recommendations?

Q12. What recommendations do you have to generally improve the [use local term]?

Q13. What was the duration/ time being you adhered to the [use local term]?

Q14. What was the engaging part of the [use local term] that you wanted to influence others?
