# Supplementary material for: Community responses to soil remediation for lead exposure reduction in Mirzapur, Bangladesh
Source: BMC Public Health. 2026 Aug 4;26:2370. doi: 10.1186/s12889-026-28814-y (PMC13476908; doi:10.1186/s12889-026-28814-y)
Supplement: Supplementary file 3 [file 12889_2026_28814_MOESM3_ESM.docx]

# Supplementary File 3: Key informant Interviews (KII) guideline

# Study title: Community Responses to Soil Remediation for Lead Exposure Reduction in Mirzapur, Bangladesh

# Key informant Interviews (KII) guideline

**Study Title: Effectiveness of soil remediation of abandoned ULAB sites, nearby household cleaning, and community awareness intervention to reduce lead exposure among children**

**IRB/ERC No.: PR-21123**

**Version Number/Date: 1.20**

*Unique ID #:*

*Location and Date:*

*Facilitator:*

*Start Time:*

*End Time:*

Goal:

- Understand perceptions of the intervention by the key informant
- Assess the acceptability and feasibility of the intervention

**Questions:**

1. **Background information of the respondent:** Name, age, sex, educational qualification, occupation, religion, contact number of the respondent, designation, role in the intervention activities
2. What do you think about the soil remediation intervention done in Gorai, Mirzapur area?
3. What were the challenges the intervention team faced during the soil remediation activities?
4. What could be the coping strategies for overcoming those challenges?
5. How was the community interaction with the intervention team during the soil remediation activities?
6. What recommendations do you have for future soil remediation activities?
7. What do you think about the lead education intervention?
8. What were the challenges the team faced during the lead education intervention?
9. What could be the coping strategies to overcome those challenges?
10. What recommendations do you have regarding the future lead education intervention?
11. For future implementation, what intervention components do you want to keep as same as this?
12. For future implementation, what intervention components do you want to change?
13. What are your suggestions for engaging the community with such intervention?
14. What are your suggestions to engage other stakeholders with such intervention?
15. What is your motivation for implementing such intervention?
